# Decreased Speech-In-Noise Understanding in Young Adults with Tinnitus

**DOI:** 10.3389/fnins.2016.00288

**Published:** 2016-06-28

**Authors:** Annick Gilles, Winny Schlee, Sarah Rabau, Kristien Wouters, Erik Fransen, Paul Van de Heyning

**Affiliations:** ^1^University Department of Otorhinolaryngology and Head and Neck Surgery, Antwerp University HospitalEdegem, Belgium; ^2^Department of Translational Neurosciences, Faculty of Medicine and Health Sciences, University of AntwerpWilrijk, Belgium; ^3^Department of Human and Social Welfare, University College GhentGhent, Belgium; ^4^University Department of Psychology, University of KonstanzKonstanz, Germany; ^5^University Department of Scientific Coordination and Biostatistics, Antwerp University HospitalEdegem, Belgium; ^6^Department of Medical Genetics, Faculty of Medicine and Health Sciences, University of AntwerpWilrijk, Belgium

**Keywords:** noise-induced tinnitus, homeostatic plasticity, young adults, recreational noise exposure, ABR, speech-in-noise testing, otoacoustic emissions, speech-in-noise understanding

## Abstract

**Objectives:** Young people are often exposed to high music levels which make them more at risk to develop noise-induced symptoms such as hearing loss, hyperacusis, and tinnitus of which the latter is the symptom perceived the most by young adults. Although, subclinical neural damage was demonstrated in animal experiments, the human correlate remains under debate. Controversy exists on the underlying condition of young adults with normal hearing thresholds and noise-induced tinnitus (NIT) due to leisure noise. The present study aimed to assess differences in audiological characteristics between noise-exposed adolescents with and without NIT.

**Methods:** A group of 87 young adults with a history of recreational noise exposure was investigated by use of the following tests: otoscopy, impedance measurements, pure-tone audiometry including high-frequencies, transient and distortion product otoacoustic emissions, speech-in-noise testing with continuous and modulated noise (amplitude-modulated by 15 Hz), auditory brainstem responses (ABR) and questionnaires.Nineteen students reported NIT due to recreational noise exposure, and their measures were compared to the non-tinnitus subjects.

**Results:** No significant differences between tinnitus and non-tinnitus subjects could be found for hearing thresholds, otoacoustic emissions, and ABR results.Tinnitus subjects had significantly worse speech reception in noise compared to non-tinnitus subjects for sentences embedded in steady-state noise (mean speech reception threshold (SRT) scores, respectively −5.77 and −6.90 dB SNR; *p* = 0.025) as well as for sentences embedded in 15 Hz AM-noise (mean SRT scores, respectively −13.04 and −15.17 dB SNR; *p* = 0.013). In both groups speech reception was significantly improved during AM-15 Hz noise compared to the steady-state noise condition (*p* < 0.001). However, the modulation masking release was not affected by the presence of NIT.

**Conclusions:** Young adults with and without NIT did not differ regarding audiometry, OAE, and ABR.However, tinnitus patients showed decreased speech-in-noise reception. The results are discussed in the light of previous findings suggestion NIT may occur in the absence of measurable peripheral damage as reflected in speech-in-noise deficits in tinnitus subjects.

## Introduction

Due to the large amount of social activities in which adolescents are exposed to high music levels such as concerts, night clubs, sports events, pubs, bars, etc., the younger population is at risk to develop noise-induced symptoms such as hearing loss, hyperacusis, and tinnitus (Smith et al., 2000; Serra et al., 2005; Beach et al., 2013) of which noise-induced tinnitus (NIT) is the symptom most frequently reported by adolescents (Widen and Erlandsson, 2004; Gilles A. et al., 2013). Tinnitus is often perceived temporarily after noise exposure, usually disappearing within a few hours. Prevalence numbers of temporary tinnitus vary from 45 to 85% depending on the definition of temporary tinnitus used by the authors (Mercier and Hohmann, 2002; Chung et al., 2005; Quintanilla-Dieck et al., 2009; Gilles A. et al., 2013). However, a significant amount of young people perceive permanent NIT for which prevalence numbers range from 3 to 15% (Widen and Erlandsson, 2004; Gilles A. et al., 2013). Several studies suggest that the amount of young people suffering from noise-induced symptoms has increased over the years (Niskar et al., 1998, 2001; Henderson et al., 2011).

Often, tinnitus is perceived in the absence of measurable hearing loss (Schaette and McAlpine, 2011). Spiral ganglion neurons are the first neural structures of the auditory system. The ganglion is almost entirely (95%) composed of type I neurons which receive synaptic input from a single inner hair cell. On the other hand, each inner hair cell forms synapses with 10–30 type I neurons (Davis and Liu, 2011). Previous research has shown that the characteristics of the sensory receptors largely determine the fundamental parameters but that the intrinsic properties of primary afferent neurons also contribute (Scroggs and Fox, 1992). Spiral ganglion neurons possess tonotopic specializations due to the tonotopically varying soma and axon diameter of putative type I neurons with the largest neurons situated toward the basal regions (Liberman and Oliver, 1984). As such, frequency coding and intensity coding is modulated by the spiral ganglion neurons by respectively gradation of the spiral ganglion neuron soma along the cochlear axis and spiral ganglion axon diameter variations around the inner hair cell circumference (Davis and Liu, 2011). Kujawa and Liberman presented an animal model showing that noise exposure caused suprathreshold response decrements (measured by auditory brainstem responses) while auditory threshold sensitivity recovered. In addition, degeneration of both the pre- and post-synaptic elements of the inner hair cell occurred throughout the basal part of the cochlea despite normal hair cell populations. It was shown that the loss of peripheral terminals of the cochlear neurons occurs almost instantly after noise exposure but that cell death and disappearance of the somata were extremely slow with a decrease of spiral ganglion cells of around 50% over a time period of 2 years (Kujawa and Liberman, 2009).

Pure-tone audiometry and otoacoustic emissions (OAEs) are well-known audiological measurements used in the daily clinical practice investigating the presence of hearing damage. Sometimes, auditory brainstem responses (ABR) are also measured in order to provide more information. However, the sensitivity of these tests in an early stage of noise damage is still under debate as it is suggested that OAEs and ABR thresholds are sensitive metrics of hair cell damage, they are insensitive to neuronal degeneration in cases loss of cochlear neurons occur in the absence of hair cell loss (Kujawa et al., 1995). Lately the focus has been on the use of speech tests for this purpose. The sensitivity of speech tests in quiet and in noise might have a higher sensitivity for subtle changes in hearing function (Jansen et al., 2013). Some studies reported that subjects with sensorineural hearing loss have worse speech reception compared to normal-hearing people during speech-in-noise testing using a steady state noise (Bacon et al., 1998; Lorenzi et al., 2006a). The use of amplitude-modulated noise during speech-in-noise testing might add important information concerning the underlying pathology in subclinical noise-induced damage. Many mechanisms are involved in the phenomenon of the so-called “masking release effect” but it is well known that “dip listening” plays an important role (Fullgrabe et al., 2006). Dip listening or “valley listening” comprises the ability to take advantage of relatively short temporal minima in the fluctuating background noise to detect important speech cues and normal hearing subjects benefit from it to a greater extent than hearing impaired persons (Festen and Plomp, 1990; Gustafsson and Arlinger, 1994; Dubno et al., 2002; Fullgrabe et al., 2006). It was suggested that this loss of ability may be attributed to an impaired temporal resolution in the hearing function of the patient group. However, studies have shown that the reduction of masking release is rather caused by impaired suprathreshold processing of the temporal and spectral domain such as abnormal coding of temporal fine-structure information and degraded frequency selectivity, rather than less-than-normal audibility (Gustafsson and Arlinger, 1994; Fullgrabe et al., 2006; Lorenzi et al., 2006a,b). Furthermore, some studies focused on the effects of tinnitus on speech reception in noise. Newman et al. showed that a group of hearing impaired tinnitus patients had significantly worse speech reception abilities compared to a control group with similar hearing impairment (Newman et al., 1994). In addition, the presence of tinnitus in the deaf ear of patients with single-sided deafness and tinnitus in the deaf ear, also affects speech reception in the non-tinnitus ear (Mertens et al., 2013). It is however still unclear whether subjects with NIT (even with normal hearing thresholds) also show decreased masking release during speech-in-noise testing. To our knowledge, amplitude-modulated noise has not yet been used in the assessment of early noise-induced hearing damage.

To date, little is known about the early signs of noise damage and it is unclear which audiological tests can detect and localize early noise-induced damage in adolescents caused by recreational noise exposure. Therefore, an extensive test protocol on a group of students was performed comprising: otoscopy, reflex measurements, tympanometry, pure-tone audiometry including high-frequency audiometry, otoacoustic emissions, speech-in-noise testing (with two types of noise masker: continuous and amplitude modulated), auditory brainstem responses, and tinnitus analysis and questionnaires in cases tinnitus was present (see Table 1). The present study aimed to reveal early signs of recreational noise damage in noise-exposed young adults by use of audiological tests available in clinical settings. It is hypothesized that young adults with tinnitus show more peripheral deficit of the auditory system compared to non-tinnitus subjects which might be expressed by poorer auditory thresholds, decreased or absent otoacoustic emissions and/or deviating ABR results. Furthermore, it might be the case that tinnitus subjects perform worse compared to the control group at a suprathreshold level reflected in poorer masking release during speech-in-modulated-noise testing.

**Table 1:** **Demographic information concerning the study subjects**.

	**Mean age (years)**	**Total of subjects (N)**	**Tinnitus (N)**	**No tinnitus (N)**
Male	23.1 ± 3.9	23	11	12
Female	23.5 ± 1.9	64	8	56

## Methods

A test protocol was developed in order to detect early-stage noise-induced damage in a young population in an early stage. The test protocol comprised the following audiological measurements: otoscopy, impedance and reflex measurements, pure-tone audiometry (including high-frequency audiometry), tinnitus analysis (in cases where tinnitus was present), otoacoustic emissions, speech-in-noise testing, auditory brainstem responses and questionnaires concerning hyperacusis and tinnitus (when present). This study was approved by the ethics committee of the University Hospital Antwerp (identification: 11/12/108). Written informed consent was obtained of all subjects. The original informed consents were archived and added to the personal medical document of the patients.

### Subjects

Subjects were recruited by sending an email for participation to all Medicine students of the University Antwerp (*N* = 650) of which 91 students replied for participation. Exclusion criteria were: the presence of pulsatile tinnitus, middle ear pathology, known neurologic diseases, history of depression, and asymmetric sensorineural hearing loss. Students had to be below the age of 30 years old and should attend parties, concerts or festivals on a weekly basis. It was however stated that students could not attend such events 2 days prior to the testing date in order to control for temporary symptoms at the time of testing. A brief and limited questionnaire concerning tinnitus presence, tinnitus etiology, and noise exposure was answered by all subjects. Nineteen students perceived permanent tinnitus defined as tinnitus present for more than 3 months at the time of testing. All tinnitus subjects indicated recreational noise exposure as the most likely causal factor of their tinnitus (next to other possibilities: occupational noise exposure, head trauma, recurrent middle ear infections, other). Students were only included when going to a party/concert for at least once a week and/or when they used personal listening devices (PLDs) several times a week at a volume level of 70% or more of the maximum capacity of the device.

In total, four students were excluded from the study due to middle ear pathology. Table 1 shows the demographic data on the test population. The following paragraphs elucidate on the methodology of the various audiological tests performed in the current protocol. ABR testing and speech-in-amplitude-modulated-noise (AM noise) was added to the test protocol in a later phase. As such, after initial testing, all participants were invited a second time for additional testing. Fifty two percent of the participants returned for the second testing moment. Table 2 provides information on the amount of subjects who underwent the various audiological measurements. In all cases the ear of each participant with the worst PTA was included for statistical analysis. In the control group (non-tinnitus group) 43 subjects showed the worst PTA score on the right side and 25 on the left side. In the tinnitus group 8 subjects showed the worst PTA score on the right side and 11 on the left side. In the latter group, the included ears also corresponded with the ear in which the tinnitus was (most loudly) perceived.

**Table 2:** **Overview of the audiological test protocol performed in subjects with and without NIT**.

**Audiological measurement**		**Number of subjects included**	
		**Tinnitus**	**No Tinnitus**
Otoscopy		19	68
Impedance and reflex measurement		19	68
Tinnitus analysis		19	n.a.
Pure tone audiometry	Classical	19	68
	High-frequency	19	68
Otoacoustic emissions	TEOAEs	19	68
	DPOAEs	19	68
Speech-in-noise testing	Steady-state noise	19	68
	AM-noise	19	23
Auditory brainstem responses		19	23
Questionnaires	Hyperacusis questionnaire	18	50
	Tinnitus questionnaire	19	n.a.
	Visual analogue scale for tinnitus loudness	19	n.a.

*HQ, hyperacusis questionnaire; TQ, Tinnitus Questionnaire and VAS-L; n.a., not applicable*.

### Pure tone audiometry

Pure tone audiometry was measured in all participating subjects according to the clinical standards (ISO 8253-1, 1989) using a two-channel Interacoustics AC-40 audiometer in a soundproof booth. Air conduction thresholds were measured at 125 Hz, 250 Hz, 500 Hz, 1 kHz, 2 kHz, 3 kHz, 4 kHz, 6 kHz, and 8 kHz. In addition, extended HFA was performed including 9, 10, 11.2, 12.5, 14, and 16 kHz. Bone conduction thresholds were measured between 250 Hz and 4 kHz.

The auditory thresholds of tinnitus and non-tinnitus subjects were compared by use of Mann–Whitney *U*-tests. A *p* ≤ 0.05 was considered as significant. Bonferroni–Holm was applied in order to correct for multiple testing. In addition, to test whether the tinnitus group contained a significantly larger number of clinically relevant outliers, the phenotype was recoded into two groups: normal (<25 dB HL) and hearing loss (≥25 dB HL), and this recoded variable was tested for association with the presence/absence of tinnitus using a Chi Square test or a Fisher's exact test (in cases where the conditions did not fit the requirements for the Chi Square test).

#### Tinnitus analysis and questionnaires

To rate the personal tinnitus disturbance two instruments were used: the Tinnitus Questionnaire (TQ) for annoyance grading and a Visual Analogue Scale (VAS) for loudness grading. The TQ is an instrument which differentiates between emotional and cognitive distress, auditory perceptual difficulties, and self-experienced intrusiveness caused by the tinnitus (Goebel and Hiller, 1994). Looking at the total score going from 0 to 84, subjects are assigned to a distress category: slight (score = 0–30, grade 1), moderate (score = 31–46, grade 2), severe (score = 47–59, grade 3), and very severe (score = 60–84, grade 4). In the present study, a Dutch validated version of the TQ was used (41). In addition, tinnitus loudness was also assessed by a VAS (VAS-L) going from 0 to 10 (0 = tinnitus is not heard at all, 10 = an extremely loud tinnitus).

The type of tinnitus was evaluated by asking whether one perceived a pulsatile or non-pulsatile tinnitus, whether the tinnitus was perceived constantly or not, unilaterally or bilaterally, and whether the tinnitus sound was a pure-tone (ringing), a noise (hissing) or a mixture of different sounds (polyphonic). Tinnitus duration was questioned during the tinnitus analysis by asking the participants for how long they have had experienced constant tinnitus.

The presence of hyperacusis was evaluated by a Flemish validated version of Khalfa's Hyperacusis Questionnaire (HQ; Khalfa et al., 2002). According to Khalfa's HQ one is diagnosed with hyperacusis when the score on the HQ is 28 or more. While validating the Dutch version of the HQ, Meeus also compared the HQ scores with other hyperacusis measurements and found one can already speak of clinically relevant hyperacusis with a score of 22 on the Flemish HQ (Meeus et al., 2010). Therefore, the score of 22 was applied as a cut-off score for the presence of hyperacusis in the present study.

### Otoacoustic emissions

Transient evoked otoacoustic emissions (TEOAEs) as well as distortion product otoacoustic emissions (DPOAEs) were measured in all subjects. TEOAEs were elicited using biphasic click sounds of 80 μs presented at an intensity level of 80 dB SPL and recorded over a frequency range of 500–4000 Hz.

DPOAEs were elicited by use of a pair of two pure tone frequencies (f1 and f2) closely spaced and presented simultaneously at a level of 55 dB SPL for f1 and 65 dB SPL for f2 (frequency ratio f2/f1 = 1.22). The largest and most robust distortion product is 2f1–f2 and can be detected in almost all normal ears.

Non-parametric tests (Mann–Whitney U) were applied to assess possible differences between tinnitus subjects and non-tinnitus subjects for TEOAEs as well as DPOAEs. A *p* ≤ 0.05 was considered as significant and the Bonferroni–Holm method was used to correct for multiple testing. In addition, to test whether the tinnitus group contained a significantly larger number of clinically relevant TEOAE and DPOAE outliers (SNR < 3 dB and SNR < 6 dB, respectively), the phenotype was recoded into two groups. For TEOAE analysis OAEs were considered as present when the SNR exceeded 3 dB and were considered as absent when the SNR was below 2.99 dB. For DPOAE analysis OAE were considered present when the SNR ≥6 dB. The presence/absence of TEOAEs/DPOAEs were tested for association with the presence/absence of tinnitus using a Pearson Chi Square test or a Fisher's exact test (in cases where the conditions did not fit the requirements for the Chi Square test).

#### Speech-in-noise testing

The Leuven Intelligibility Sentence Test (LIST; Van Wieringen and Wouters, 2008), a Dutch sentence test, was applied. The LIST consists of 35 lists of 10 sentences that are a reflection of daily communication and are of equivalent difficulty. An adaptive procedure is used with the noise at a fixed level of 65 dB SPL. The procedure starts at a signal-to-noise ratio (SNR) of 0 dB meaning that speech and noise are presented equally loud (65 dB SPL). Subsequently, the intensity level within a list of sentences is varied in steps of 2 dB adaptively in a 1-down (when the keywords in the sentence are correctly repeated), 1-up (when the keywords in the sentence are incorrectly repeated) procedure to determine the 50% correct identification point which is called the speech reception threshold (SRT), expressed in dB SNR. Before starting the actual procedure, one list was performed as a training list. Speech reception was calculated as the mean SNR obtained by the subject. For example, a score of −5 dB SNR means that the speech could be 5 dB quieter than the noise which is fixed at 65 dB SPL.

During the presentation of the sentences two kinds of noise masker were applied. A first noise masker was a steady-state noise spectrally matched with the long-term average speech spectrum so that the SNR would be, on average, approximately equal at all frequencies (Van Wieringen and Wouters, 2008). For the other masker used during the second testing moment, the stationary noise was amplitude-modulated by 15 Hz with a modulation depth of 100%, from now on referred to as AM noise.

Subjects were seated in a quiet room and the sentences were presented monaurally through headphones. The sentences were played directly from a computer using software interface TigerSpeech Technology (2012) and passed through an audiometer. Sentence levels were adjusted by the software during adaptive testing depending whether the keywords in the sentences were repeated correctly or incorrectly. Also the noise was played from the same software passing through the audiometer and was presented to the ipsilateral ear at a fixed level of 65 dB SPL. The levels of speech and noise were calibrated by a licensed company prior to the commencement of the experiment.

In order to test for normal distributions of the components, Shapiro–Wilk test of normality was applied and Q-Q plots were visually inspected. All variables were normally distributed (*p* > 0.05 for Shapiro–Wilk) in both groups. Consequently, parametric tests (student-*t*) were performed in order to find differences between speech reception in tinnitus and non-tinnitus subjects for steady-state noise as well as AM noise. In addition, parametric tests were performed to reveal possible differences in performance between speech reception during stationary noise and during AM noise for tinnitus as well as non-tinnitus subjects separately. Bonferroni–Holm correction was applied to correct for multiple testing.

In addition, a repeated measures ANOVA analysis was performed in order to see whether there was a different masking release effect of AM-noise for tinnitus subjects and non-tinnitus subjects when compared to the steady-state noise condition.

### Auditory brainstem responses

Auditory brainstem responses (ABR) were recorded in a subset of tinnitus and non-tinnitus patients with the Bio-Logic Auditory Evoked Potentials system (version 6.2.1.1) and a Bio-Logic Navigator Pro® interface. The skin was prepared by use of a Nuprep gel in order to lower the skin impedance which had to be below 5 kOhm and inter-electrode impedance had to be below 2 kOhm. Electrodes were placed on both mastoids and on the high forehead with the common electrode on the lower forehead. Subjects laid down on a comfortable bed and the light was subdued. Subjects were also instructed to keep the eyes closed during the measurements and to minimize all muscle activity as much as possible.

100 μs-duration clicks were presented with alternating polarity through ER-3A earphones at a rate of 31.0 stimuli/s and a level of 80 dBnHL (= dB normalized hearing level). Contralateral white noise masking was applied with an intensity of 55 dBnHL. The signal was high pass filtered from 100 Hz and low pass filtered from 3000 Hz. Artifact rejection was set at 23.8 μV and a maximum of 2000 averages was recorded. In order to obtain the best possible outcome of the ABR testing, all recordings were repeated 3 times to ensure reproducibility.

ABR component amplitudes (baseline-to-peak) and latencies were determined by visual inspection of the waveforms I–V. Wave V is the most robust waveform in an adult population. Other waveforms may not always occur or be accurately identified by the clinician. In addition, latency ratios I–III, I–V, and III–V were calculated as well as the interpeak ratios I–III, I–V, and III–V.

Previous research has shown that the exact matching for pure-tone audiometry thresholds (with maximum differences of 5 dB HL) of groups is a prerequisite in order to be able to correctly analyze ABR data (Gu et al., 2012). As such, two separate analyses were performed. The first analysis was based on the data matched at a group level. Subsequently, a more precise matching was performed where every tinnitus subject was age- and gender-matched as well as matched until the level of 5 dB HL for pure-tone thresholds from 1 to 4 kHz (as this is the maximum frequency spectrum tested by the ABR). Therefore, in the second analysis, less participants were included to ensure the exact matching and only 10 pairs of perfectly matched subjects were obtained. The latter analysis was performed in addition to the group analysis in order to obtain reliable results. If Shapiro–Wilk test for normality showed normal data distributions, parametric testing was used.

## Results

Nineteen subjects had permanent tinnitus, corresponding with 22% of the sample. Noise exposure was briefly evaluated by a short questionnaire. All students with tinnitus attributed their tinnitus to recreational noise exposure. Party/concert attendance of students was approximately once a week. Sixty four percent of the control group and 52% of the tinnitus group attended a musical venue more than once a week.

Details on the tinnitus type, side and severity are shown in Tables 3, 4. Most young adults perceived a bilateral, pure-tone tinnitus with only limited tinnitus distress (on average a grade 1 on the TQ). The mean tinnitus duration was 2 years (*SD* = 1.2 years) and none of the tinnitus subjects reported tinnitus from childhood. The HQ was filled out by most subjects showing a clear difference between non-tinnitus and tinnitus subjects. Tinnitus subjects scored significantly higher on the HQ (mean = 15.39; *SD* = 6.65) compared to non-tinnitus subjects (mean = 7.71; *SD* = 7.96; *p* = 0.001). Only few subjects were diagnosed with hyperacusis though this symptom was relatively more prevalent in the tinnitus group. In the non-tinnitus group (*N* = 68) three subjects had a score >22 on the HQ while already four subjects of the tinnitus group (*N* = 19) had scores above 22. The sample of students with hyperacusis was too small to perform further statistical analysis.

**Table 3:** **Distribution of tinnitus type and tinnitus side characteristics assessed in the tinnitus group**.

**Tinnitus characteristic**		***N* subjects**
Type	Pure-tone	13
	Noise	6
	Polyphonic	0
Side	Unilateral	2
	Bilateral	16
	Central	1

**Table 4:** **Mean scores for the TQ and VAS-L, including standard deviations for the tinnitus subjects**.

**Tinnitus questionnaires**	**Mean**	***SD***
TQ score	27.72	15.23
VAS-L score	5.44	2.46

**Figures 1, 2:**
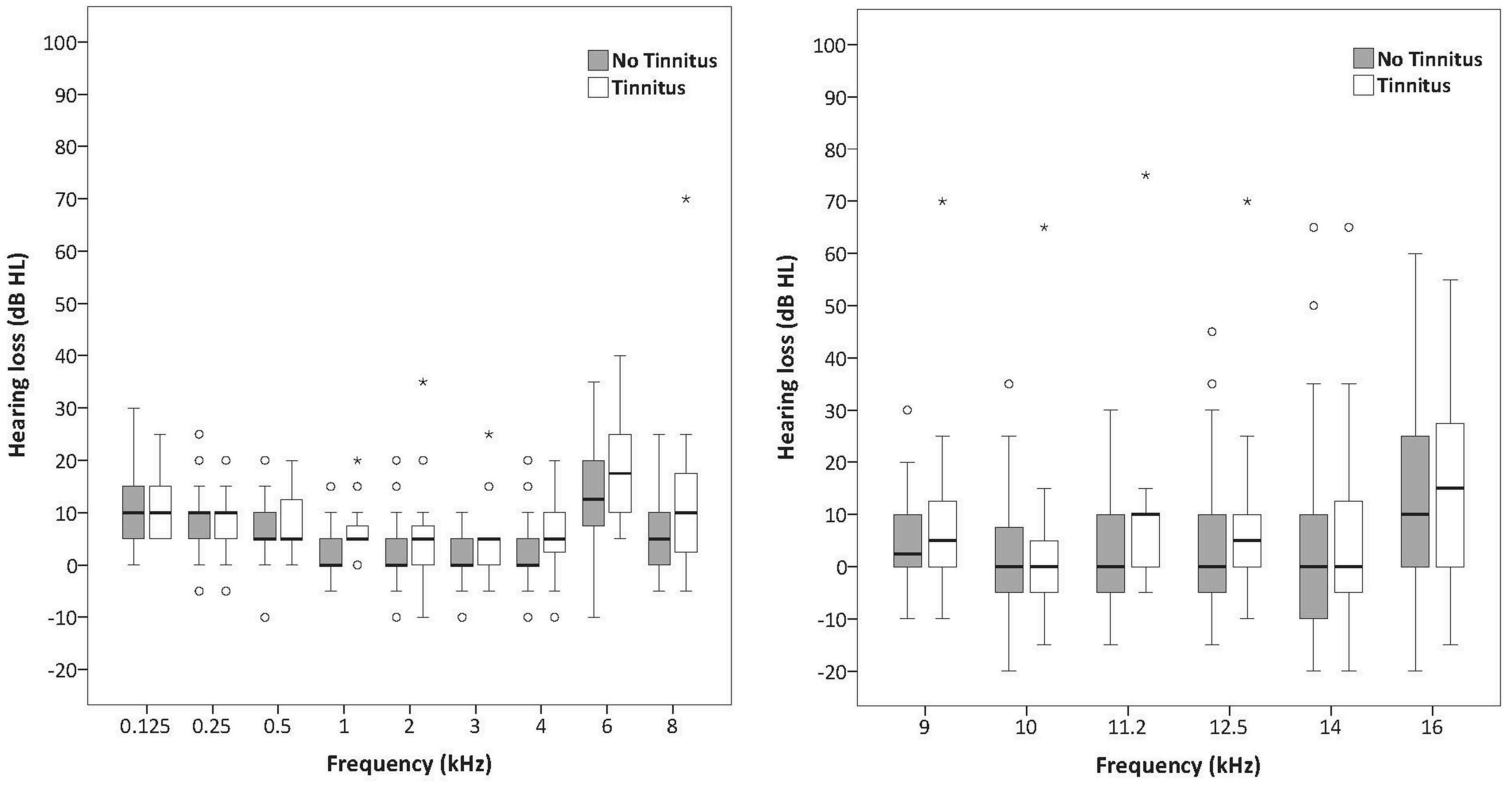
**Boxplots representing pure tone audiometry for conventional frequencies (125–8 kHz) and high frequencies (9–16 kHz) for tinnitus subjects and non-tinnitus subjects**. The box length is the interquartile range (IQR). A circle represents outliers with values between 1.5 and 3 box lengths from the upper or lower edge of the box. Asterisks represent extreme outliers (more than 3 times the IQR).

Figures 1, 2 illustrate the median and the variability for the conventional audiometry and the HFA respectively as well as the differences between the tinnitus and non-tinnitus subjects. No significant differences in hearing thresholds between tinnitus subjects and non-tinnitus subjects were apparent (see Table 5). Some outliers could be noted where the hearing thresholds exceeded 25 dB HL which no longer could be considered as normal hearing. It was investigated whether such outliers were more prevalent in the tinnitus group compared to the controls by use of a Fisher's exact test. Within the power of the current study, there is no indication that the tinnitus group contained a significantly larger fraction of outliers compared to the control group. More insight into the audiometric data is provided in the Supplementary Materials section.

**Table 5:** **Audiometric differences between the tinnitus and the non-tinnitus group for all measured frequencies**.

**Audiometric frequency**	***p*-value (uncorrected)**	***p*-value (corrected)**
125 Hz	0.52	1.00
250 Hz	0.75	1.00
500 Hz	0.52	1.00
1 kHz	<0.01	0.06
2 kHz	0.24	1.00
3 kHz	0.34	1.00
4 kHz	0.02	0.25
6 kHz	0.07	0.86
8 kHz	0.19	1.00
9 kHz	0.76	1.00
10 kHz	0.95	0.95
11.2 kHz	0.16	1.00
12.5 kHz	0.33	1.00
14 kHz	0.26	1.00
16 kHz	0.64	1.00

*Bonferroni-Holm correction was applied for multiple testing correction but uncorrected values are provided as well*.

**Figures 3, 4:**
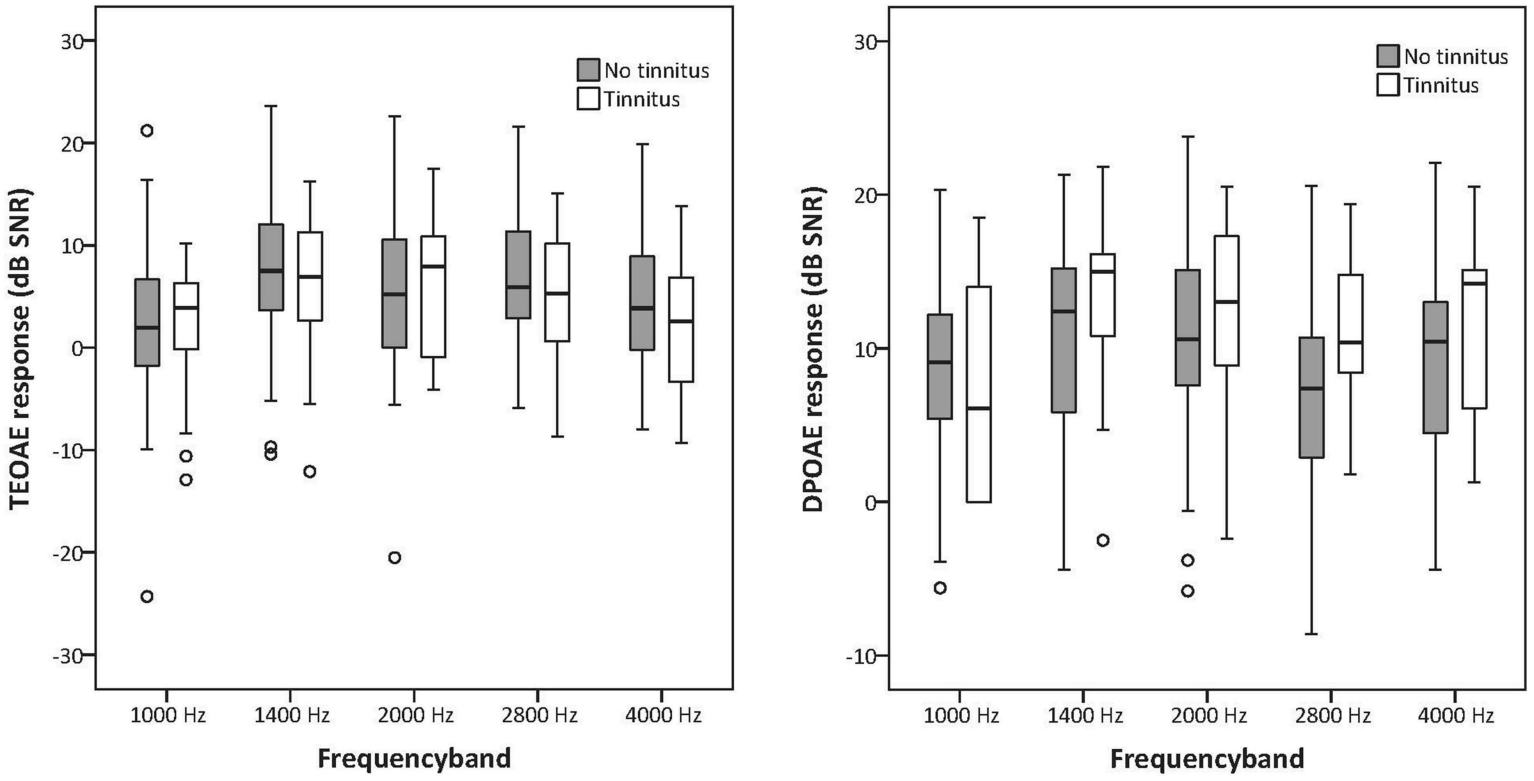
**Boxplots representing TEOAE and DPOAE data for tinnitus subjects and controls**. The box length is the interquartile range (IQR). A circle represents outliers with values between 1.5 and 3 box lengths from the upper or lower edge of the box.

TEOAE and DPOAE were compared between tinnitus and non-tinnitus subjects for each frequency band. Tables 6, 7 summarize the otoacoustic emissions data which is also plotted in Figures 3, 4. No significant differences in OAE strength were found between groups for the measured TEOAE as well as DPOAE frequency bands. By the use Mann–Whitney *U*-tests to compare OAEs between the tinnitus and the control group it is likely to miss crucial differences between the two groups. In particular, clinically relevant outliers in the case group may be missed, since nonparametric tests transform the observed values into ranks. Therefore, additional dichotomizing was performed where TEOAEs were considered as present when the SNR was equal to or exceeded 3 dB SNR and DPOAEs were considered as present when ≥6 dB SNR. Chi square tests and Fisher's exact tests did not show—within the power of the current study—significant differences in the prevalence of present/absent OAEs in both groups. More insight into the latter analyses is provided in the Supplementary Material section.

**Table 6:** **Differences in TEOAE band-frequency strength [signal-to-noise ratio (SNR)] between tinnitus and non-tinnitus subjects**.

**TEOAE band-frequency (kHz)**	**Median TEOAE strength (dB SNR)**		***p*-value (uncorrected)**	***p*-value (corrected)**
	**Tinnitus**	**No tinnitus**		
1	3.90	1.95	0.83	1.0
1.4	6.90	7.50	0.98	0.98
2	7.90	5.20	0.86	1.0
2.8	5.30	5.90	0.63	1.0
4	2.60	3.85	0.25	1.0

*Uncorrected p-values are given and were corrected for multiple testing by use of Bonferroni–Holm*.

**Table 7:** **Differences in DPOAE band-frequency strength between tinnitus and control subjects**.

**DPOAE band-frequency (kHz)**	**Median DPOAE strength (dB SNR)**		***p*-value (uncorrected)**	***p*-value**
	**Tinnitus**	**No tinnitus**		
1	6.10	9.10	0.80	0.80
1.4	15.00	12.40	0.15	0.45
2	13.00	10.60	0.28	0.57
2.8	10.40	7.40	0.01	0.07
4	14.20	10.45	0.10	0.41

*Uncorrected p-values are given and were corrected for multiple testing by use of Bonferroni–Holm*.

**Figure 5:**
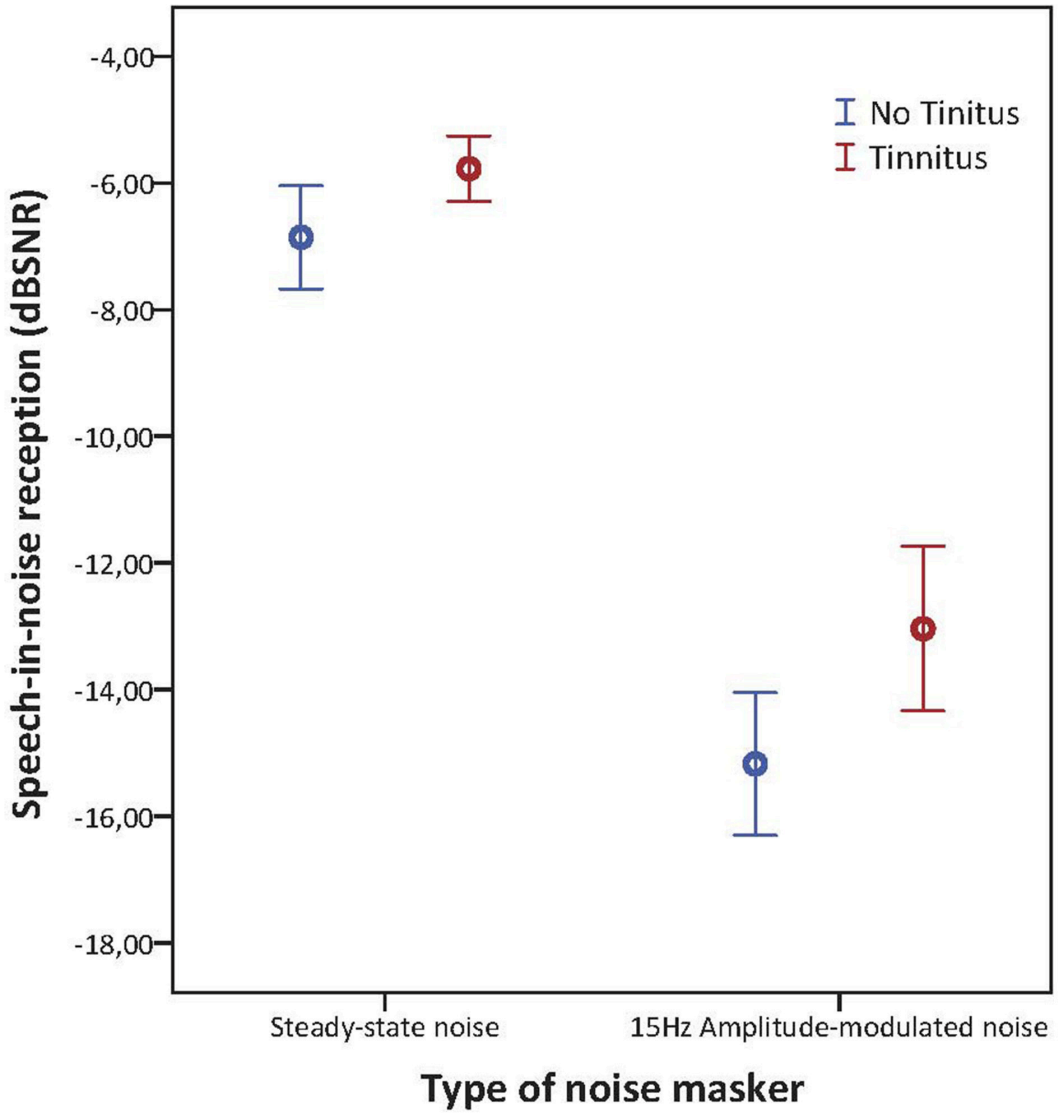
**Error barchart illustrating speech-in-noise reception in steady state noise and AM-15 Hz noise of tinnitus and non-tinnitus subjects**. The bars represents the 95% confidence interval and the standard error is depicted by the whiskers.

Concerning the speech-in-noise testing, tinnitus subjects had significantly worse SRT scores compared to non-tinnitus subjects for sentences embedded in steady-state noise (mean SRT scores, respectively −5.77 and −6.90 dB SNR; *p* = 0.025) as well as for sentences embedded in 15 Hz AM-noise (mean SRT scores, respectively −13.04 and −15.17 dB SNR; *p* = 0.013) as illustrated in Figure 5. In the repeated measures ANOVA the between subject effect was “group” and the within subject effect was “noise type.” In addition, the interaction between noise type and group was investigated. Significant effects were shown for group and speech-in-noise (*p* < 0.001) but no interaction effect was apparent (*p* = 0.162) meaning that the increase of masking release when going from steady-state noise to AM noise were quite similar for both groups with the difference that tinnitus subjects had a worse starting point. Also, a logistic regression was performed in order to explain the variance provided by speech-in-noise testing in NIT subjects. By use of this analysis it was confirmed that speech-in-noise testing by use of steady-state noise was worse in NIT subjects (*p* = 0.018) as well as in AM-15 Hz noise (*p* = 0.011). In addition, it was found that speech-in-noise testing explains 40% of the variance (Nagelkerke *R*^2^ = 0.403).

Visual inspection of the ABR waveforms were performed. Wave V is the most robust wave in an adult population. Other waves may not always occur or be accurately identified by the clinician by use of visual inspection. Independent student's *t*-tests were performed in order to reveal differences between the tinnitus and control group for the latency and/or amplitude of the different ABR waveforms. Table 8 provides an overview of the mean latencies, amplitudes, interpeak latencies/amplitudes, standard deviations, and the outcome of the independent student's *t*-test between tinnitus subjects and controls. After correction for multiple testing by Bonferroni–Holm, no statistical differences could be shown between tinnitus subjects and controls within the power of the current study.

**Table 8:** **overview of the detectability of each wave (= N), mean values for wave latency (in ms) and amplitude (in μV) and standard deviations**.

**Variable**	**Group**	**N**	**Mean**	***SD***	***p*-value for independent *t*-test (uncorrected)**	***p*-value for independent *t*-test (corrected)**
**LATENCY**						
Wave I	Controls	23	1.57	0.11	0.63	1.00
	Tinnitus	19	1.60	0.17		
Wave II	Controls	17	2.74	0.17	0.57	1.00
	Tinnitus	7	2.78	0.11		
Wave III	Controls	23	3.75	0.24	0.17	1.00
	Tinnitus	19	3.67	0.18		
Wave IV	Controls	9	5.01	0.16	0.07	0.98
	Tinnitus	5	4.81	0.19		
Wave V	Controls	23	5.51	0.23	0.88	1.00
	Tinnitus	19	5.53	0.33		
**INTERPEAK LATENCY**						
Wave I–III	Controls	23	2.18	0.17	0.16	1.00
	Tinnitus	19	2.06	0.28		
Wave III–V	Controls	23	1.78	0.12	0.05	0.75
	Tinnitus	19	1.91	0.31		
Wave I–V	Controls	23	3.94	0.18	0.54	1.00
	Tinnitus	19	3.98	0.22		
**AMPLITUDE**						
Wave I	Controls	23	0.10	0.08	0.12	1.00
	Tinnitus	19	0.14	0.08		
Wave II	Controls	17	0.06	0.06	0.09	1.00
	Tinnitus	7	0.11	0.07		
Wave III	Controls	23	0.20	0.10	0.18	1.00
	Tinnitus	19	0.26	0.15		
Wave IV	Controls	10	0.10	0.06	0.38	1.00
	Tinnitus	5	0.07	0.05		
Wave V	Controls	23	0.23	0.13	0.14	1.00
	Tinnitus	19	0.18	0.07		
**INTERPEAK AMPLITUDE**						
Wave I–III	Controls	23	0.10	0.15	0.77	1.00
	Tinnitus	19	0.12	0.15		
Wave III–V	Controls	23	0.02	0.14	0.04	0.64
	Tinnitus	19	0.08	0.17		
Wave I–V	Controls	23	0.13	0.19	0.08	1.00
	Tinnitus	19	0.04	0.12		

*Corrected and uncorrected p-values are shown for the independent samples t-test*.

For the second analysis, for which a very thorough matching was applied (see ABR description in the Methods Section), paired student's *t*-tests were performed but again did not show any statistical different ABR results between tinnitus and non-tinnitus subjects concluding the ABR data did not differ between groups in the present dataset.

## Discussion and conclusions

### Audiological tests assessing the peripheral pathway

The current study examined a group of 87 recreationally noise-exposed university students. In total 19 students, corresponding to 22% of the study population, experienced NIT which was present for more than 3 months at the time of testing. Earlier epidemiological studies on noise-induced symptoms in Belgian adolescents has shown a high prevalence of NIT in this population in line with the current findings (Gilles et al., 2012; Gilles A. et al., 2013; Degeest et al., 2014). The present study compared test results of various audiological tests between the students with and without tinnitus. The testing consisted of pure-tone audiometry (including high frequencies), otoacoustic emissions, ABR, and speech-in-noise testing (with steady-state noise and amplitude-modulated noise). No significant differences in audiometric thresholds between tinnitus and control subjects could be observed. Most had normal hearing thresholds and the prevalence of outliers (thresholds >25 dB HL) was equal in both groups. Furthermore, TEOAEs, DPOAEs, and ABRs did not show significant differences between the groups. However, speech-in-noise reception was significantly decreased in tinnitus subjects. The following paragraphs discuss these findings in the light of previous findings suggesting the presence of (noise-induced) tinnitus may occur in the absence of measurable peripheral damage and might cause more central plasticity than expected.

It has been suggested that extended high frequency audiometry (HFA) testing might reveal cochlear damage at higher frequencies than investigated by a conventional audiogram (Yildirim et al., 2010; Mehrparvar et al., 2011). Concerning the application of HFA in young people exposed to recreational noise, only limited research has been performed so far. However, it has been shown that, when no signs of noise-induced hearing damage can be detected on the conventional audiometry (125 Hz to 8 kHz), hearing thresholds at the higher frequencies (9–16 kHz) can be significantly increased (Sulaiman et al., 2013). However, the current study shows that adolescents with NIT do not necessarily have decreased hearing thresholds on conventional nor high-frequency audiometry. Also, audiometric outliers (>25 dB HL) were not more prevalent in the tinnitus group compared to controls indicating that there were no significant differences in hearing thresholds between the groups. This fact raises questions concerning the applicability of pure-tone audiometry as an assessment tool for the evaluation of early noise-induced damage. Besides HFA, evidence for the clinical use of OAEs in the early detection of noise-induced damage is growing (Sliwinska-Kowalska and Kotylo, 1997; Prasher and Sulkowski, 1999). It has been suggested that OAEs might reveal outer hair cell damage before it is reflected in the audiogram (Sliwinska-Kowalska and Kotylo, 1997, 2001). Sulaiman et al. showed increased high-frequency thresholds and decreased DPOAE amplitudes in a group of PLD users in the absence of measurable hearing loss between 125 Hz and 8 kHz (Sulaiman et al., 2013). Also McKee and Stephens showed decreased OAEs in tinnitus subjects with normal hearing (McKee and Stephens, 1992). In the current study, TEOAEs as well as DPOAEs were performed in all students but no significant differences between tinnitus and non-tinnitus subjects could be observed. Similar results were obtained in a younger population in a recent study by Sanchez et al. assessing 168 adolescents by use of pure-tone audiometry (250 Hz to 16 kHz), TEOAEs and DPOAEs. 28.6% of the sample experienced permanent tinnitus, 28% sporadic tinnitus and the remaining 43.4% did not have tinnitus. No significant differences were observed between the groups regarding audiometric thresholds and TEOAEs/DPOAEs (Sanchez et al., 2015). Considering the present results as well as findings from previous studies, the use of OAEs in noise-exposed subjects is still under debate. Possibly OAEs might render more information in cases of acute acoustic trauma with temporary threshold shift in order to more precisely investigate specific frequency regions such as the 3–6 kHz region (Buchler et al., 2012). However, as a tool for early noise-induced hearing damage screening, the overall results of studies are rather inconclusive at this point (Shupak et al., 2007), suggesting that the addition of OAE measurements to the golden standard of audiometry, is not sufficient in detecting early-staged noise-induced hearing damage.

The current study did not find any differences in ABR results between tinnitus subjects and controls. This is in line with the study by Barnea et al. who performed HFA and ABR testing on a tinnitus group with normal hearing sensitivity in the range of 125 Hz to 8 kHz compared to an age- and gender-matched control group. Similar to the present study, high-frequency and ABR audiometric data did not differ between the considered groups (Barnea et al., 1990). Although not found in the present study, an I–V amplitude ratio alteration was previously reported. Schaette and McAlpine found reduced wave I potentials in normal-hearing female tinnitus subjects but normal amplitude of the more centrally generated wave V. The authors concluded that the deviation of wave I, which is generated by the primary auditory nerve fibers, provides direct evidence for “hidden hearing loss” that manifests as reduced neural output coming from the cochlea followed by renormalization of neural response magnitude within the brainstem reflected by normal wave V amplitudes (Schaette and McAlpine, 2011). Similar results were obtained for male subjects in another study where tinnitus subjects also showed reduced wave I amplitudes but, in addition, enhanced wave V reflecting elevated input to the inferior colliculi. Also elevated I–III and I/V amplitude ratios were apparent implicating disproportionally high activity in spherical bush cells in the ventral cochlear nucleus (Gu et al., 2012). Intergender differences exist in auditory brainstem response amplitudes and latencies (Durrant et al., 1990). Therefore, the current study also investigated possible differences in ABR results within male and female subjects for tinnitus subjects vs. controls (see also Supplementary Material). No gender differences were apparent in the current data set. However, it has to be pointed out that the control group contained more female subjects than males and therefore these results should be interpreted with caution.

### Peripheral vs. central deficits

The deteriorating effects of hearing loss on speech reception in noise have been thoroughly investigated in previous research (Festen and Plomp, 1990; Bacon et al., 1998; Peters et al., 1998; Bernstein and Grant, 2009; Rhebergen et al., 2010). The decreased release of masking that occurs in hearing impaired subjects could only be partly explained by reduced audibility (Bacon et al., 1998). The ability to correctly detect the temporal fine structure of speech (Fullgrabe et al., 2006; Lorenzi et al., 2006a) and to process spectral details (Nelson et al., 2003; Nelson and Jin, 2004), seems critical for dip-listening. As loss of ability to use temporal and spectral cues in speech is highly associated with sensorineural hearing loss (Bacon et al., 1998) the question arises why speech reception in noise is also sometimes decreased in normal hearing subjects in the absence of hearing loss (Middelweerd et al., 1990). In the present study, tinnitus subjects had significantly worse speech reception in steady-state noise as well as in AM noise. Ryu et al. also found decreased speech perception ability (in quiet as well as in noise) in normal-hearing tinnitus subjects compared to controls (Ryu et al., 2012) in line with earlier findings by Huang et al. (2007). A recent study including tinnitus subjects with and without hearing loss and a control group, measured spectral and temporal resolution as well as speech-in-noise reception. No significant differences between tinnitus subjects and controls were found concerning spectral and temporal abilities. However, SRT scores were significantly worse in tinnitus subjects (Moon et al., 2015). It is discussed that normal temporal and spectral resolution in tinnitus subjects reflect the undisturbed functionality of OHCs in the cochlea. In case of cochlear damage, the basilar membrane response would be more linear and broadly tuned resulting in reduced compression and broadening of the auditory filters which would negatively affect both frequency selectivity and temporal resolution (Moore and Glasberg, 1986; Oxenham and Bacon, 2003). The findings of decreased SRT scores in the absence of temporal/spectral resolution deficits imply that tinnitus can occur without OHC damage and might depend more on plastic changes in the central auditory system (Moon et al., 2015). The present results confirm the latter findings as no differences in peripheral functioning, tested by pure-tone audiometry, and OAEs, could be observed. In this study however, spectral and temporal resolution was not specifically tested but speech-in-amplitude-modulated noise testing gives a robust idea of temporal encoding when listening in the gaps. Speech reception in AM noise was significantly worse in tinnitus patients compared to controls but to the same extent as speech reception in steady-state noise. Hence, no additional temporal deficits are apparent in the tinnitus group. It can be suggested that speech reception testing in AM noise does not add useful information to the classical speech reception test in steady-state noise. However, in line with previous findings by Moon et al. the suggestion can be made that the decreased speech reception in subjects with NIT, in the absence of measurable cochlear lesions, might be due to a more central deficit.

The presence of tinnitus in the absence of measurable cochlear hearing loss, as is the case in the present study, forms a serious challenge to the model of cortical hyperactivity. However, Kujawa and Liberman earlier reported that in an animal experiment 50–60% of the auditory nerve fibers in the high-frequency region of the cochlea were deafferented after mild acoustic trauma without any permanent auditory threshold elevation suggesting that normal hearing thresholds do not necessarily exclude the possibility of cochlear damage (Kujawa and Liberman, 2009). It is posited that acoustic overexposure can produce a rapid and irreversible loss of cochlear nerve peripheral terminals on IHCs and a slow degeneration of spiral ganglion cells, despite full recovery of cochlear thresholds and no loss of IHCs or OHCs (Kujawa and Liberman, 2009; Lin et al., 2011). The phenomenon of noise-induced cochlear neuronal degeneration in mice independent of auditory threshold changes, described by Kujawa and Liberman is recently described as “cochlear synaptopathy” (Liberman et al., 2015). Recently, it has been shown that a deficiency in pejvakin protein can cause exceptional vulnerability to sound as pejvakin deficient cochleae exhibit features of marked oxidative stress and impaired antioxidant defenses (Delmaghani et al., 2015). Although aforementioned studies are animal studies, a study by Weisz et al. supported the presence of deafferentiation in the absence of audiometrically detectable hearing loss in humans with tinnitus by use of the Threshold Equalizing Noise test (TEN test; Weisz et al., 2006). However, these results were not confirmed in one of our previous studies (Gilles A. D. et al., 2013). The authors believe that in case of presence of cochlear synaptopathy or pejvakin deficiency in the tinnitus subjects, ABR data of the tinnitus subjects would have shown amplitude and latency decrements which were not apparent in the present study. Not-withstanding, the results of the current study does not exclude the possible interference of (currently unmeasurable) peripheral damage in NIT.

However, it can also be argued that when all audiometric findings show normal results in NIT subjects, retrocochlear deficits are present which result into the tinnitus percept on one hand but may also influence performance on a broader scale. The neural correlates of tinnitus have been described as auditory as well as non-auditory (Langers and Melcher, 2011; Langguth et al., 2012; Vanneste and De Ridder, 2012) suggesting that speech performance may also be altered as a consequence of cortical reorganization. It was shown by a previous study that brainstem responses evoked by speech in subjects with speech message decoding difficulties, may reveal miscoding in subcortical structures as an origin (Kraus and Nicol, 2005). In addition, deficiencies in higher-order cortical networks have been found in tinnitus subjects with normal hearing thresholds (Melcher et al., 2009).

## Conclusions and future research

The current study examined a group of 87 recreationally noise-exposed university students. The argument can be made that, ideally, also a group of non-exposed students should be included in order to investigate their audiological characteristics and compare them to the subject groups with occasional recreational noise exposure. However, as recreational noise exposure is an undeniable part of the current society, it is rather impossible to find young subjects who did not have any kind of noise exposure during their lifespan. Therefore, the decision was made to only include young adults with a certain amount of noise exposure. Noise exposure was evaluated by use of a very limited questionnaire broadly evaluating the current frequency of social events with high music levels as well as noise exposure caused by PLDs. However, noise exposure during the lifespan was not assessed meaning that, inevitably, there were possibly (small to large) differences in the total amount of noise exposure. As such, it cannot be ruled out by the current analysis that tinnitus subjects did not have more noise exposure. Nevertheless, the scope of the present study was not to investigate nor calculate noise exposure in adolescents but the focus was on the effects of recreational noise on the audiological characteristics. As some students experienced NIT, which can be considered as a symptom of noise damage, this symptom was used to make a comparison in audiological characteristics between tinnitus and non-tinnitus subjects.

No peripheral lesions could be observed in the current study evaluated by pure-tone audiometry, OAEs, and ABR. Speech-in-noise testing however was significantly decreased in tinnitus subjects possibly suggesting more centrally located deficits in tinnitus subjects. In addition, it can be said that cortical reorganization may occur due to frequent exposure to recreational noise exposure in the absence of any measurable peripheral hearing loss. The present article underlines the need for further testing besides the conventional audiometry. The sensitivity of pure-tone audiometry as well as OAE measurements might be insufficient to detect peripheral noise-induced damage at an early stage and one must interpret normal outcome results with this technique with caution. The authors like to mention the theory of “homeostatic plasticity” described by Gourévitch et al. (2014). These authors showed that reversible noise-induced threshold shifts may mask progressive underlying neuropathology that likely has profound long-term consequences on auditory processing. A normal audiogram is considered as the “golden standard.” However, although the peripheral parts of the auditory system seem to be functioning normal (expressed by normal audiogram and OAE measurements in the present study), substantial changes may occur in the auditory brain post noise exposure. The mechanism of “central gain” at the level of the auditory brainstem, or more cortically located, causes initially a decrease in synaptic efficacy in central parts of the auditory system in the noise-exposed frequency region. Long-lasting changes in synaptic efficacy after prolonged noise exposure could affect the expression of inhibition (Gourévitch et al., 2014). Gourevitch et al. provided evidence that the peripheral auditory system can be harmed without decreased auditory thresholds and that long-lasting disturbance of the excitation-inhibition balance in the central auditory system may eventually lead to cortical reorganization as a result of homeostatic plasticity (Gourévitch et al., 2014). Homeostatic plasticity mechanisms may regulate a central gain mechanism, and tinnitus might be a side-effect of these changes (Norena and Farley, 2013; Brotherton et al., 2015). In addition, it has to be noted that hyperacusis was more prevalent in the tinnitus sample than in the controls. Psychoacoustic measurements in the tinnitus subjects might have rendered additional, important information and it can be considered as a limitation of the study that these measurements were not performed. Further research is required in order to investigate the role of homeostatic plasticity in recreational noise exposure.

In conclusion, speech-in-noise testing forms a reliable and clinically feasible technique to assess noise-induced damage in patients with normal peripheral function but with complaints of NIT. The present study shows promising results concerning the use of speech-in-noise testing in detection of noise-induced damage in adolescents. The use of amplitude-modulated noise however, requires more research in order to further investigate the mechanism of homeostatic plasticity in recreational noise exposure as well as the peripheral involvement. Furthermore, late-auditory evoked potentials might provide additional information on higher-order cortical processing of (speech) sounds (Joos et al., 2014) and might therefore also render useful information in normal-hearing tinnitus subjects.

## Author contributions

AG: This author has substantially contributed to the design of the study, the acquisition, analysis, and interpretation of the data, is the main author for drafting the manuscript. WS: This author has substantially contributed to the interpretation of the study and the writing of the manuscript. He agrees to be accountable for all aspects of the work. SR: This author has substantially contributed to the collection of the data and the final version of the manuscript. KW: This author has substantially contributed to the analysis of the data and reviewed the final draft very carefully. EF: This author has substantially contributed to the analysis of the data and reviewed the final draft very carefully. PV: This author was involved in the design, analysis and interpretation of the study and has contributed to the writing of the manuscript. All authors gave final approval of this version to be published as such and agrees to be accountable for all aspects of the work in ensuring that questions related to the accuracy or integrity of any part of the work are appropriately investigated and resolved.

## Funding

The main author is an employee of the University Hospital Antwerp and as such, this research is funded by the hospital. No external funding bodies were involved in the current study.

### Conflict of interest statement

The authors declare that the research was conducted in the absence of any commercial or financial relationships that could be construed as a potential conflict of interest.
