# Supplementary material for: Decreased Speech-In-Noise Understanding in Young Adults with Tinnitus
Source: Front Neurosci. 2016 Jun 28;10:288. doi: 10.3389/fnins.2016.00288 (PMC4923253; doi:10.3389/fnins.2016.00288)
Supplement: Supplementary file 1 [file DataSheet1.DOCX]

**Supplementary Material**

1. **Variance of variables**

To test if the variance in the tinnitus group was different from the variance in the control group, a Levene’s test for homogeneity of variances was carried. Table 1 provides the uncorrected p-values for all the tested parameters.

| **Variable** | **p-value** |
| --- | --- |
| *Auditory thresholds* |  |
| 125 Hz | 0.408 |
| 250Hz | 1.000 |
| 500 Hz | 0.132 |
| 1 kHz | 0.059 |
| 2 kHz | 0.069 |
| 3 kHz | 0.081 |
| 4 kHz | 0.362 |
| 6 kHz | 0.456 |
| 8 kHz | 0.035 |
| 9 kHz | 0.015 |
| 10 kHz | 0.552 |
| 11.2 kHz | 0.937 |
| 12.5 kHz | 0.637 |
| 14 kHz | 0.531 |
| 16 kHz | 0.665 |
|  |  |
| *TEOAE frequencyband* |  |
| 1000 Hz | 0.773 |
| 1400 Hz | 0.465 |
| 2000 Hz | 0.688 |
| 2800 Hz | 0.505 |
| 4000 Hz | 0.113 |
|  |  |
| *DPOAE frequencyband* |  |
| 1000 Hz | 0.021 |
| 1400 Hz | 0.611 |
| 2000 Hz | 0.666 |
| 2800 Hz | 0.906 |
| 4000 Hz | 0.506 |
|  |  |
| *Speech-in-noise* |  |
| Steady-state noise | 0.116 |
| Amplitude-modulated noise | 0.958 |
|  |  |
| *ABR latenties* |  |
| Wave I | 0.058 |
| Wave II | 0.122 |
| Wave III | 0.520 |
| Wave IV | 0.555 |
| Wave V | 0.536 |
| Interpeak latency I-III | 0.582 |
| Interpeak latency III-V | 0.028 |
| Interpeak latency I-V | 0.409 |
|  |  |
| *ABR amplitudes* |  |
| Wave I | 0.943 |
| Wave II | 0.782 |
| Wave III | 0.312 |
| Wave IV | 0.854 |
| Wave V | 0.010 |
| Interpeak amplitude I-III | 0.439 |
| Interpeak amplitude III-V | 0.876 |
| Interpeak amplitude I-V | 0.054 |

*Table 1: Levene’s test for homogeneity of variances.*

1. **Further information on statistical analyses on audiometric data**

To test whether the tinnitus group contained a significantly larger number of clinically relevant audiometric outliers, the phenotype was recoded into two groups: normal hearing (< 25dB HL) and hearing loss (≥25 dB HL), and this recoded variable was tested for association with the presence/absence of tinnitus using a Pearson Chi Square test or a Fisher’s exact test (in cases where the conditions did not fit the requirements for the Chi Square test). To provide more insight into the data for the reader, following crosstabs are provided to show the amount of outliers (≥25 dB HL) in each group for every pure-tone threshold.

|  | | | **Group** | |
| --- | --- | --- | --- | --- |
|  |  |  | **Controls** | **tinnitus** |
| **125 Hz** | **< 25 dB HL** | Count | 67 | 19 |
|  |  | % within group | 98.5% | 100% |
|  | **> 25 dB HL** | Count | 1 | 0 |
|  |  | % within group | 1.5% | 0.0% |
| **Fisher’s Exact Test p-value**  **= 1.00** | | | | |

|  | | | **Group** | |
| --- | --- | --- | --- | --- |
|  |  |  | **Controls** | **tinnitus** |
| **250 Hz** | **< 25 dB HL** | Count | 68 | 19 |
|  |  | % within group | 100% | 100% |
|  | **> 25 dB HL** | Count | 0 | 0 |
|  |  | % within group | 0.0% | 0.0% |
| **Chi Square Test could not be performed (all subjects had thresholds < 25 dB HL)** | | | | |

|  | | | **Group** | |
| --- | --- | --- | --- | --- |
|  |  |  | **Controls** | **tinnitus** |
| **500 Hz** | **< 25 dB HL** | Count | 68 | 19 |
|  |  | % within group | 100% | 100% |
|  | **> 25 dB HL** | Count | 0 | 0 |
|  |  | % within group | 0.0% | 0.0% |
| **Chi Square Test could not be performed (all subjects had thresholds < 25 dB HL)** | | | | |

|  | | | **Group** | |
| --- | --- | --- | --- | --- |
|  |  |  | **Controls** | **tinnitus** |
| **1000 Hz** | **< 25 dB HL** | Count | 68 | 19 |
|  |  | % within group | 100% | 100% |
|  | **> 25 dB HL** | Count | 0 | 0 |
|  |  | % within group | 0.0% | 0.0% |
| **Chi Square Test could not be performed (all subjects had thresholds < 25 dB HL)** | | | | |

|  | | | **Group** | |
| --- | --- | --- | --- | --- |
|  |  |  | **Controls** | **tinnitus** |
| **2000 Hz** | **< 25 dB HL** | Count | 68 | 18 |
|  |  | % within group | 100% | 94.7% |
|  | **> 25 dB HL** | Count | 0 | 1 |
|  |  | % within group | 0.0% | 5.3% |
| **Fisher’s Exact Test p-value**  **= 0.218** | | | | |

|  | | | **Group** | |
| --- | --- | --- | --- | --- |
|  |  |  | **Controls** | **tinnitus** |
| **3000 Hz** | **< 25 dB HL** | Count | 68 | 19 |
|  |  | % within group | 100% | 100% |
|  | **> 25 dB HL** | Count | 0 | 0 |
|  |  | % within group | 0.0% | 0.0% |
| **Chi Square Test could not be performed (all subjects had thresholds < 25 dB HL)** | | | | |

|  | | | **Group** | |
| --- | --- | --- | --- | --- |
|  |  |  | **Controls** | **tinnitus** |
| **4000 Hz** | **< 25 dB HL** | Count | 68 | 19 |
|  |  | % within group | 100% | 100% |
|  | **> 25 dB HL** | Count | 0 | 0 |
|  |  | % within group | 0.0% | 0.0% |
| **Chi Square Test could not be performed (all subjects had thresholds < 25 dB HL)** | | | | |

|  | | | **Group** | |
| --- | --- | --- | --- | --- |
|  |  |  | **Controls** | **tinnitus** |
| **6000 Hz** | **< 25 dB HL** | Count | 62 | 15 |
|  |  | % within group | 91.2% | 83.3% |
|  | **> 25 dB HL** | Count | 6 | 3 |
|  |  | % within group | 8.8% | 16.7% |
| **Pearson Chi Square Test p-value = 0.33** | | | | |

|  | | | **Group** | |
| --- | --- | --- | --- | --- |
|  |  |  | **Controls** | **tinnitus** |
| **8000 Hz** | **< 25 dB HL** | Count | 68 | 18 |
|  |  | % within group | 100% | 94.7% |
|  | **> 25 dB HL** | Count | 0 | 1 |
|  |  | % within group | 0.0% | 5.3% |
| **Fisher’s Exact Test p-value = 0.22** | | | | |

|  | | | **Group** | |
| --- | --- | --- | --- | --- |
|  |  |  | **Controls** | **tinnitus** |
| **9000 Hz** | **< 25 dB HL** | Count | 65 | 18 |
|  |  | % within group | 95.6% | 94.7% |
|  | **> 25 dB HL** | Count | 3 | 1 |
|  |  | % within group | 4.4% | 5.3% |
| **Fisher’s Exact Test p-value = 1.00** | | | | |

|  | | | **Group** | |
| --- | --- | --- | --- | --- |
|  |  |  | **Controls** | **tinnitus** |
| **10 000 Hz** | **< 25 dB HL** | Count | 67 | 18 |
|  |  | % within group | 98.5% | 94.7% |
|  | **> 25 dB HL** | Count | 1 | 1 |
|  |  | % within group | 1.5% | 5.3% |
| **Fisher’s Exact Test p-value = 0.40** | | | | |

|  | | | **Group** | |
| --- | --- | --- | --- | --- |
|  |  |  | **Controls** | **tinnitus** |
| **11 200 Hz** | **< 25 dB HL** | Count | 66 | 18 |
|  |  | % within group | 98.5% | 94.7% |
|  | **> 25 dB HL** | Count | 2 | 1 |
|  |  | % within group | 3.0% | 5.3% |
| **Fisher’s Exact Test p-value = 0.53** | | | | |

|  | | | **Group** | |
| --- | --- | --- | --- | --- |
|  |  |  | **Controls** | **tinnitus** |
| **12 500 Hz** | **< 25 dB HL** | Count | 65 | 18 |
|  |  | % within group | 95.5% | 94.7% |
|  | **> 25 dB HL** | Count | 3 | 1 |
|  |  | % within group | 4.5% | 5.3% |
| **Fisher’s Exact Test p-value = 1.00** | | | | |

|  | | | **Group** | |
| --- | --- | --- | --- | --- |
|  |  |  | **Controls** | **tinnitus** |
| **14 000 Hz** | **< 25 dB HL** | Count | 63 | 17 |
|  |  | % within group | 92.5% | 89.5% |
|  | **> 25 dB HL** | Count | 5 | 2 |
|  |  | % within group | 7.5% | 10.5% |
| **Fisher’s Exact Test p-value = 0.65** | | | | |

|  | | | **Group** | |
| --- | --- | --- | --- | --- |
|  |  |  | **Controls** | **tinnitus** |
| **16 000 Hz** | **< 25 dB HL** | Count | 53 | 14 |
|  |  | % within group | 77.6% | 73.7% |
|  | **> 25 dB HL** | Count | 15 | 5 |
|  |  | % within group | 22.4% | 26.3% |
| **Pearson Chi Square Test p-value = 0.72** | | | | |

1. **Further information on statistical analyses on otoacoustis emission data**

To test whether the tinnitus group contained a significantly larger number of clinically relevant TEOAE and DPOAE outliers (SNR < 3dB and SNR < 6dB respectively), the phenotype was recoded into two groups. For TEOAE analysis OAEs were considered as present when the SNR exceeded 3 dB and were considered as absent when the SNR was below 2.99 dB. For DPOAE analysis OAE were considered present when the SNR ≥ 6 dB. The presence/absence of TEOAEs/DPOAEs were tested for association with the presence/absence of tinnitus using a Pearson Chi Square test or a Fisher’s exact test (in cases where the conditions did not fit the requirements for the Chi Square test). In order to provide more insight into the data for the reader, following crosstabs are provided to show the amount of outliers in each group for all OAE-frequency bands.

**TEOAE**

|  | | | **Group** | |
| --- | --- | --- | --- | --- |
|  |  |  | **Controls** | **tinnitus** |
| **1000 Hz frequency band** | **< 3 dB HL** | Count | 37 | 8 |
|  |  | % within group | 54.4% | 42.1% |
|  | **≥ 3 dB HL** | Count | 31 | 11 |
|  |  | % within group | 45.6% | 57.9% |
| **Pearson Chi Square Test p-value**  **= 0.34** | | | | |

|  | | | **Group** | |
| --- | --- | --- | --- | --- |
|  |  |  | **Controls** | **tinnitus** |
| **1400 Hz frequency band** | **< 3 dB HL** | Count | 15 | 6 |
|  |  | % within group | 22.1% | 31.6% |
|  | **≥ 3 dB HL** | Count | 53 | 13 |
|  |  | % within group | 77.9% | 68.4% |
| **Pearson Chi Square Test p-value**  **= 0.40** | | | | |

|  | | | **Group** | |
| --- | --- | --- | --- | --- |
|  |  |  | **Controls** | **tinnitus** |
| **2000 Hz frequency band** | **< 3 dB HL** | Count | 25 | 7 |
|  |  | % within group | 36.8% | 36.8% |
|  | **≥ 3 dB HL** | Count | 43 | 12 |
|  |  | % within group | 63.2% | 63.2% |
| **Pearson Chi Square Test p-value**  **= 0.10** | | | | |

|  | | | **Group** | |
| --- | --- | --- | --- | --- |
|  |  |  | **Controls** | **tinnitus** |
| **2800 Hz frequency band** | **< 3 dB HL** | Count | 18 | 6 |
|  |  | % within group | 26.5% | 31.6% |
|  | **≥ 3 dB HL** | Count | 50 | 13 |
|  |  | % within group | 73.5% | 68.4% |
| **Pearson Chi Square Test p-value**  **= 0.66** | | | | |

|  | | | **Group** | |
| --- | --- | --- | --- | --- |
|  |  |  | **Controls** | **tinnitus** |
| **4000 Hz frequency band** | **< 3 dB HL** | Count | 28 | 10 |
|  |  | % within group | 41.2% | 52.6% |
|  | **≥ 3 dB HL** | Count | 40 | 9 |
|  |  | % within group | 58.8% | 47.4% |
| **Pearson Chi Square Test p-value**  **= 0.37** | | | | |

**DPOAE**

|  | | | **Group** | |
| --- | --- | --- | --- | --- |
|  |  |  | **Controls** | **tinnitus** |
| **1000 Hz frequency band** | **< 6 dB HL** | Count | 16 | 9 |
|  |  | % within group | 23.5% | 47.4% |
|  | **≥ 6 dB HL** | Count | 52 | 10 |
|  |  | % within group | 76.5% | 52.6% |
| **Pearson Chi Square Test p-value**  **= 0.042** | | | | |

|  | | | **Group** | |
| --- | --- | --- | --- | --- |
|  |  |  | **Controls** | **tinnitus** |
| **1400 Hz frequency band** | **< 6 dB HL** | Count | 16 | 3 |
|  |  | % within group | 23.5% | 15.8% |
|  | **≥ 6 dB HL** | Count | 52 | 16 |
|  |  | % within group | 76.5% | 84.2% |
| **Pearson Chi Square Test p-value**  **= 0.47** | | | | |

|  | | | **Group** | |
| --- | --- | --- | --- | --- |
|  |  |  | **Controls** | **tinnitus** |
| **2000 Hz frequency band** | **< 6 dB HL** | Count | 10 | 3 |
|  |  | % within group | 14.7% | 17.6% |
|  | **≥ 6 dB HL** | Count | 58 | 14 |
|  |  | % within group | 85.3% | 82.4% |
| **Pearson Chi Square Test p-value**  **= 0.76** | | | | |

|  | | | **Group** | |
| --- | --- | --- | --- | --- |
|  |  |  | **Controls** | **tinnitus** |
| **2800 Hz frequency band** | **< 6 dB HL** | Count | 25 | 3 |
|  |  | % within group | 36.8% | 17.6% |
|  | **≥ 6 dB HL** | Count | 43 | 14 |
|  |  | % within group | 63.2% | 82.4% |
| **Pearson Chi Square Test p-value**  **= 0.13** | | | | |

|  | | | **Group** | |
| --- | --- | --- | --- | --- |
|  |  |  | **Controls** | **tinnitus** |
| **4000 Hz frequency band** | **< 6 dB HL** | Count | 18 | 4 |
|  |  | % within group | 26.5% | 21.1% |
|  | **≥ 6 dB HL** | Count | 50 | 15 |
|  |  | % within group | 73.5% | 78.9% |
| **Pearson Chi Square Test p-value**  **= 0.63** | | | | |

1. **Further information on statistical analyses on ABR data**

Intergender differences exist in auditory brainstem response amplitudes and latencies. Therefore, the current study also investigated possible differences in ABR results within male and female subjects for tinnitus subjects versus controls.

| **Variable** | **Group** | **N** | **Mean** | **SD** | **p-value for independent t-test (uncorrected)** | **p-value for independent t-test (corrected)** |
| --- | --- | --- | --- | --- | --- | --- |
| *Latency* | | | | | | |
| Wave I | Controls | 21 | 1.58 | 0.12 | 0.65 | 1.00 |
|  | Tinnitus | 8 | 1.60 | 0.13 |  |  |
| Wave II | Controls | 15 | 2.74 | 0.63 | 0.42 | 1.00 |
|  | Tinnitus | 4 | 2.81 | 0.06 |  |  |
| Wave III | Controls | 21 | 3.76 | 0.25 | 0.50 | 1.00 |
|  | Tinnitus | 8 | 3.70 | 0.06 |  |  |
| Wave IV | Controls | 7 | 5.01 | 0.16 | 0.13 | 1.00 |
|  | Tinnitus | 3 | 4.73 | 0.21 |  |  |
| Wave V | Controls | 21 | 5.51 | 0.23 | 0.30 | 1.00 |
|  | Tinnitus | 8 | 5.35 | 0.39 |  |  |
| *Interpeak latency ratio* | | | | | | |
| Wave I-III | Controls | 21 | 2.18 | 1.80 | 0.21 | 1.00 |
|  | Tinnitus | 8 | 2.10 | 0.08 |  |  |
| Wave III-V | Controls | 21 | 1.76 | 0.12 | 0.69 | 1.00 |
|  | Tinnitus | 8 | 1.78 | 0.15 |  |  |
| Wave I-V | Controls | 21 | 3.94 | 0.18 | 0.45 | 1.00 |
|  | Tinnitus | 8 | 3.87 | 0.20 |  |  |
| *Amplitude* | | | | | | |
| Wave I | Controls | 21 | 0.09 | 0.08 | 0.39 | 1.00 |
|  | Tinnitus | 8 | 0.12 | 0.09 |  |  |
| Wave II | Controls | 15 | 0.05 | 0.07 | 0.27 | 1.00 |
|  | Tinnitus | 4 | 0.11 | 0.09 |  |  |
| Wave III | Controls | 21 | 0.21 | 0.10 | 0.30 | 1.00 |
|  | Tinnitus | 8 | 0.27 | 0.22 |  |  |
| Wave IV | Controls | 8 | 0.10 | 0.07 | 0.52 | 1.00 |
|  | Tinnitus | 3 | 0.07 | 0.07 |  |  |
| Wave V | Controls | 21 | 0.23 | 0.13 | 0.47 | 1.00 |
|  | Tinnitus | 8 | 0.20 | 0.08 |  |  |
| *Interpeak amplitude ratio* | | | | | | |
| Wave I-III | Controls | 21 | 0.11 | 0.15 | 0.66 | 1.00 |
|  | Tinnitus | 8 | 0.15 | 0.21 |  |  |
| Wave III-V | Controls | 21 | 0.03 | 0.14 | 0.19 | 1.00 |
|  | Tinnitus | 8 | 0.06 | 0.21 |  |  |
| Wave I-V | Controls | 21 | 0.14 | 0.19 | 0.40 | 1.00 |
|  | Tinnitus | 8 | 0.08 | 0.12 |  |  |

Table 2: ABR data for female tinnitus subjects versus female control subjects. Overview of the detectability of each wave (= N), mean values for wave latency (in ms) and amplitude (in µV) and standard deviations. Corrected and uncorrected p-values are shown for the independent samples t-test.

| **Variable** | **Group** | **N** | **Mean** | **SD** | **p-value for independent t-test (uncorrected)** | **p-value for independent t-test (corrected)** |
| --- | --- | --- | --- | --- | --- | --- |
| *Latency* | | | | | | |
| Wave I | Controls | 2 | 1.55 | 0.03 | 0.78 | 1.00 |
|  | Tinnitus | 11 | 1.59 | 0.19 |  |  |
| Wave II | Controls | 2 | 2.72 | 0.33 | 0.94 | 1.00 |
|  | Tinnitus | 3 | 2.74 | 0.15 |  |  |
| Wave III | Controls | 2 | 3.70 | 0.06 | 0.69 | 1.00 |
|  | Tinnitus | 11 | 3.63 | 0.23 |  |  |
| Wave IV | Controls | 2 | 4.99 | 0.23 | 0.84 | 1.00 |
|  | Tinnitus | 2 | 4.94 | 0.06 |  |  |
| Wave V | Controls | 2 | 5.55 | 0.21 | 0.52 | 1.00 |
|  | Tinnitus | 11 | 5.65 | 0.20 |  |  |
| *Interpeak latency ratio* | | | | | | |
| Wave I-III | Controls | 2 | 2.15 | 0.09 | 0.77 | 1.00 |
|  | Tinnitus | 11 | 2.06 | 0.38 |  |  |
| Wave III-V | Controls | 2 | 1.86 | 0.15 | 0.60 | 1.00 |
|  | Tinnitus | 11 | 2.00 | 0.37 |  |  |
| Wave I-V | Controls | 2 | 3.40 | 0.23 | 0.70 | 1.00 |
|  | Tinnitus | 11 | 4.06 | 0.21 |  |  |
| *Amplitude* | | | | | | |
| Wave I | Controls | 2 | 0.18 | 0.06 | 0.62 | 1.00 |
|  | Tinnitus | 11 | 0.15 | 0.07 |  |  |
| Wave II | Controls | 2 | 0.09 | 0.01 | 0.74 | 1.00 |
|  | Tinnitus | 3 | 0.11 | 0.06 |  |  |
| Wave III | Controls | 2 | 0.17 | 0.06 | 0.29 | 1.00 |
|  | Tinnitus | 11 | 0.25 | 0.09 |  |  |
| Wave IV | Controls | 2 | 0.08 | 0.06 | 0.76 | 1.00 |
|  | Tinnitus | 2 | 0.06 | 0.06 |  |  |
| Wave V | Controls | 2 | 0.16 | 0.13 | 0.95 | 1.00 |
|  | Tinnitus | 11 | 0.16 | 0.07 |  |  |
| *Interpeak amplitude ratio* | | | | | | |
| Wave I-III | Controls | 2 | 0.01 | 0.00 | 0.21 | 1.00 |
|  | Tinnitus | 11 | 0.09 | 0.12 |  |  |
| Wave III-V | Controls | 2 | 0.01 | 0.18 | 0.43 | 1.00 |
|  | Tinnitus | 11 | 0.09 | 0.12 |  |  |
| Wave I-V | Controls | 2 | 0.02 | 0.18 | 0.81 | 1.00 |
|  | Tinnitus | 11 | 0.01 | 0.12 |  |  |

Table 3: ABR data for male tinnitus subjects versus male control subjects. Overview of the detectability of each wave (= N), mean values for wave latency (in ms) and amplitude (in µV) and standard deviations. Corrected and uncorrected p-values are shown for the independent samples t-test.
